# Host Adaptation of Soybean Dwarf Virus Following Serial Passages on Pea (*Pisum sativum*) and Soybean (*Glycine max*)

**DOI:** 10.3390/v9060155

**Published:** 2017-06-21

**Authors:** Bin Tian, Frederick E. Gildow, Andrew L. Stone, Diana J. Sherman, Vernon D. Damsteegt, William L. Schneider

**Affiliations:** 1 Department of Plant Pathology, The Pennsylvania State University, University Park, PA 16802, USA; btian@ksu.edu (B.T.); feg2@psu.edu (F.E.G.); 2 USDA-ARS Foreign Disease Weed Science Research Unit, Fort Detrick, MD 21702, USA; andrew.stone@ars.usda.gov (A.L.S.); diana.sherman@ars.usda.gov (D.J.S.); verndd@hotmail.com (V.D.D.); 3 Department of Plant Pathology, Kansas State University, Manhattan, KS 66506, USA

**Keywords:** Soybean Dwarf Virus, trade-off effect, aphid transmission, soybean aphid, adaptation, genome sequencing

## Abstract

Soybean Dwarf Virus (SbDV) is an important plant pathogen, causing economic losses in soybean. In North America, indigenous strains of SbDV mainly infect clover, with occasional outbreaks in soybean. To evaluate the risk of a US clover strain of SbDV adapting to other plant hosts, the clover isolate SbDV-MD6 was serially transmitted to pea and soybean by aphid vectors. Sequence analysis of SbDV-MD6 from pea and soybean passages identified 11 non-synonymous mutations in soybean, and six mutations in pea. Increasing virus titers with each sequential transmission indicated that SbDV-MD6 was able to adapt to the plant host. However, aphid transmission efficiency on soybean decreased until the virus was no longer transmissible. Our results clearly demonstrated that the clover strain of SbDV-MD6 is able to adapt to soybean crops. However, mutations that improve replication and/or movement may have trade-off effects resulting in decreased vector transmission.

## 1. Introduction

Soybean Dwarf Virus (SbDV), first identified as a pathogen of soybean plants in northern Japan in 1969, is capable of causing serious yield losses in soybean [1]. The host range of SbDV is largely limited to members of the Fabaceae except for a few species in *Chenopodiaceae* and *Polemoniaceae* [2]. Currently, four distinct strains of SbDV are recognized in Japan based on sequencing, symptomatology in infected soybean plants and aphid vector specificity [3]. The yellowing strains, SbDV-YS and SbDV-YP, cause severe interveinal chlorosis, rugosity and thickening of leaves in soybeans. The dwarfing strains, SbDV-DS and SbDV-DP, cause stunting with shortened internodes and brittle curled leaves. The SbDV-YS and -DS strains are transmitted specifically by the aphid *Aulacorthum solani* Kaltenbach, while SbDV-YP and -DP strains are transmitted by both *Acyrthosiphon pisum* Harris, and *Nearctaphis bakeri* Cowen. In the United States, SbDV-like isolates were identified in asymptomatic white clover (*Trifolium repens*), symptomatic red clover (*Trifolium pratense*), yellow sweet clover (*Melilotus officinalis*), and subterranean clover (*Trifolium subterraneum* L.) from 11 mid-western and eastern states [4]. In 2000, it was reported that soybean (*Glycine max* L Merr.) crops were infected by a SbDV-Y strain in Virginia [5]. During 2003, dwarfing strains of SbDV emerged in soybean plants accompanied by the soybean aphid, *Aphis glycines* Matsumura, in Wisconsin [6]. A survey of soybean diseases in northern Illinois also identified two SbDV-D strains [7]. Recently, the presence of mixed infections of both D and Y strains were confirmed in the eastern United States [8], and several U.S. isolates were found to be transmitted by *A. glycines* [9] Currently, there are no SbDV resistant commercial cultivars available. More recently, it has been found that the *Rsdv1* quantitative trait loci gene was responsible for resistance of an Indonesian cultivar ‘Wilis’ [10], but the mechanism of such gene is still unclear [11]. It raises the interesting question that if the rapid evolved plant virus could emerge in a new environment and cause disease outbreak in the soybean field. 

SbDV is a luteovirus, with a positive sense single-stranded RNA genome. The genomic RNAs of SbDV range from 5.7 to 5.9 kb, comprising five open reading frames (ORFs) and three untranslated regions (UTRs). ORF 1 and ORF 2 encode the replication-related proteins. ORF 3 encodes the 22-kiloDalton (kDa) major coat protein (CP) that is the major component of the capsid, and ORF 4, which is nested within ORF 3, putatively encodes a movement protein. ORF 5 encodes a 65–88 kDa Read-Through Protein (RTP), the minor capsid protein, formed by the in-frame translational readthrough of the ORF3 stop codon. ORFs 3, 4 and 5 on the 3′-end of SbDV show similarities to genus *Polerovirus*, while ORFs 1 and 2 show similarities to the genus *Luteovirus* [12,13].

Plant viruses have several mechanisms to generate genetic diversity both within and between species. Plant RNA viruses have highly error prone replication mechanisms, which result in numerous mutations and diverse populations. Mutation, recombination, and re-assortment are three major forces driving the evolution of viruses, and these forces generate diversity in viral genomes, providing variants to adapt to different environments [14]. Viral emergence and adaptation to new hosts and/or new host resistances is one of the highest impact effects of plant virus evolution [15]. Even minor changes in viral genomes can result in significant phenotypic effects. One study indicated that the site-specific mutant on amino acid 27 of NIaPro of Papaya Ringspot Virus (PRSV) determined the ability of infecting the host papaya [16]. Another study on Soybean Mosaic Virus (SbMV) showed that precise mutations in the *HC-Pro* gene were essential for virulence on different resistant genotypes of soybeans [17]. However, there may be limitations to the amount of host adaptation a plant virus can tolerate. Adaptation to a new host could result in fitness losses in original hosts because mutations beneficial in the new host might be detrimental to infection of the original hosts, a phenomenon called the trade-off effect [18]. For example, the Tobacco Etch Virus (TEV) adapted to a new host, pepper, as indicated by an increase in virulence and virus accumulation, whereas the viral fitness in the natural tobacco host was decreased [19]. In the case of SbDV, a trade-off effect that increases fitness in soybeans while decreasing fitness in clover could have significant epidemiological impacts, as clover is the overwintering reservoir for the virus.

Reports indicate that SbDV is widely prevalent in clover in North America [20]. There are currently only limited reports of SbDV emergence in soybean fields [6,21], despite the high profile and acreage of this important crop. It is quite probable that SbDV is undergoing adaptation in the transition from clover to soybean. The risks of SbDV outbreaks in soybean would seem to be higher following the recent introduction of the soybean aphid (*A. glycines*), and the establishment of this potential SbDV-vector species on soybean crops. Therefore, a better understanding of the capacity for SbDV host adaptation is important to evaluate potential risk of SbDV epidemics in commercial soybean crops in the U.S. The objectives of this study are to evaluate the SbDV fitness in different plant hosts and identify critical mutations of SbDV selected by such new host adaptations.

## 2. Materials and Methods

### 2.1. Plants, Viruses, and Aphid Vector

For this study, Puget pea (*Pisum sativum* cv. Puget), soybeans (*Glycine max* cv. Williams 82), and white clover (*Trifolium repens*) seedlings were used for host serial transmissions. A white clover infected with a SbDV-Y isolate (MD6) was collected from a field survey in Maryland. SbDV-MD6 was maintained in white clover from the time of field collection until the beginning of the host passaging. In the aphid transmission experiments, *A. pisum* (pea aphid) was used as the aphid vector for pea passages, and *N. bakeri* was used as the vector for soybean passages. Pea aphids were reared on caged faba bean plants (*Vicia faba* L.), and clover aphids were reared on red clovers (*Trifolium pratense* L.) in an aphid rearing room maintained at 25 °C with 24 h photoperiod.

### 2.2. Serial Transmission Assays

For the transmission experiment (Figure S1), aphids acquired viruses by feeding on detached leaves of infected white clover for 24 h. Then, the aphids were transferred to healthy pea or soybean seedlings for 5 days. At the same time, three healthy seedlings of each plant species were fed on by healthy aphids as a negative control. For *A. pisum* on peas, 3 aphids or 10 aphids per plant were used in the different serial transmission lines. For *N. bakeri* on soybeans, approximately 30 aphids per plant were used in all serial transmission lines. Fifteen plant seedlings were used in each passage. The number of aphids used per plant on each host species was determined by preliminary transmission efficiency tests (data not shown). After inoculation, the presence of SbDV in inoculated plants was determined using ELISA commercially available kits according to manufacturer directions (Agdia, Elkhart, IN, USA). Detached leaves from all infected plants were pooled to serve as the source for the next transmission. Each passage line was continued through eight passages, or until transmission failed. SbDV-MD6 was transmitted four times by both aphids in the original host, white clover, as a positive control for transmission, and as a control to determine whether subsequent effects were related to host shifts or merely to passaging. Serial transmission experiments were conducted with two replications, and the transmission efficiency between initial and final passage was analyzed by chi-squared test from Minitab 16 (Minitab Inc., State College, PA, USA).

### 2.3. Plant Height and Fresh Weight Measurements and Statistical Analysis

To compare healthy and infected plants from various passages, plant height was measured at 25 days post-inoculation (d.p.i.). The fresh weight of pea stems and leaves was tested by weighing plants at 30 d.p.i. The statistical significance of differences in plant height and fresh weight between non-infected and infected plants, and among passages was subjected to *t*-test analysis from Minitab 16 (Minitab Inc., State College, PA, USA).

### 2.4. RNA Extraction and Real Time RT-PCR

In each transmission experiment, leaf samples from all infected plants were collected from every passage individually, and flash-frozen in liquid nitrogen and stored at −80 °C for RNA extraction. The leaf tissue from each infected plant was randomly cut by a leaf punch with 1 cm diameter to grind in tubes with liquid nitrogen, and 3 discs were used for extracting total RNA. Total RNAs of pooled samples were isolated using the RNeasy Mini Kit (Qiagen Science, Louisville, KY, USA) according to manufacturer specifications, and diluted in 50 µL buffer. SbDV RNA was amplified using first strand cDNA synthesis as follows: 2 µL of total nucleic acid was added to 1× first strand buffer (Invitrogen, Carlsbad, CA, USA), 10 mM dithiothreitol 300 µM dNTP mix, 40 U SuperScript II (Invitrogen, Carlsbad, CA, USA), and 200 nM SbDV reverse primers (Figure S2) to cover the full length of the SbDV genome. The reaction was incubated at 42 °C for 1 h. Amplification followed by using 1 µL of the first strand reaction for the quantitative polymerase chain reaction (qPCR). The primers used in the qPCR assay were 1880UR (5′-CAT TTA TTG GCT ATT ATC TTC C-3′) and 1548YF (5′-CAA AGT TGG TCC AAG GAC CC-3′), and the fluorescently labeled probe was 1728UP (5′-GAT AGC ACC CAG GTT GAT ATG T-3′). The reaction was run in a SmartCycler (Cepheid, Sunnyvale, CA, USA) machine with the following conditions: 10 min 95 °C denaturing followed by 45 cycles of 95 °C for 15 s and 60 °C for 1 min. The assay positive result threshold was calculated by the SmartCycler program using 10 standard deviations above background fluorescence. To determine the effectiveness of quantification, a fragment of SbDV-MD6 was cloned into a plasmid with a T7 RNA polymerase promoter. This plasmid was linearized and used to make in vitro transcripts, which served both as positive controls for quatitative reverse transcription-PCR (qRT-PCR) and as a directly quantifiable RNA template to establish standard curves. The assay consistently detected less than 1 femtogram (fg) of SbDV RNA transcripts. Regression analysis of the SbDV standard curve demonstrated that the assay was highly efficient (*R*^2^ = 0.998) indicating that it could be used to estimate SbDV-RNA concentration (data not shown). The number of the copies of SbDV-RNA was estimated by the formula $\left(\mathrm{pg}\;\times \;6.023\;\times \;10^{6}\right)/3.74$. The statistical analysis of virus titers was done with one-way ANOVA, and a significant difference was tested using Fisher’s method.

### 2.5. Back Inoculation Assays

Various selected passages from the serial transmission on peas were used as a source for back inoculation assays, to test the ability of aphids to transmit the pea- or soybean-adapted SbDV back into the clover host. Only the first and the final soybean passage was used as a source for back inoculation assay. The two aphid vectors, *A. pisum* or *N. bakeri*, were fed on infected pea or soybean leaves, respectively, for 24 h, and then transferred to healthy white clover seedlings. The pea and soybean passaged SbDV-MD6 was transmitted to six and 10 healthy clover plants, respectively, with 6 plants for positive controls (*A. pisum* transmitting SbDV from pea to pea, and *N. bakeri* transmitting source SbDV from clover to clover). All plants were tested by ELISA and total RNA was isolated for sequencing from positive samples.

### 2.6. Sequencing and Analysis

Total RNA was extracted as previously described. RT-PCR products were generated for the final passages from each of the passage lines using specific primer pairs designed to cover the entire SbDV genome (Figure S2). The PCR products were directly sequenced with both directions to determine the consensus sequences (most common sequence). The complete genome sequences of tested passages were sequenced and compared to the viral genome from the original clover isolate using Clustal_X sequence analysis in software Geneious R9 (Biomatters Limited., Newark, NJ, USA). Consistent mutations (mutations that occurred in both passage lines, and were maintained through to the end of passaging) between the source isolates and alternative host passages were identified, and effects on amino acid content of proteins were determined. The populations from back assays were sequenced using the same approach to look for reversion of the potential adaptive mutations. The *d*_N_/*d*_S_ ratio was calculated using MEGA 6.0 [22] with the Nei–Gojobori method.

## 3. Results

### 3.1. Serial Passages and Vector Transmission Efficiency on Peas and Soybeans

To investigate the potential ability of SbDV-MD6 to adapt to other alternative hosts, peas (cv. Puget) and soybeans (cv. Williams82) were used as plant hosts for serial transmissions of SbDV-MD6 by *A. pisum* and *N. bakeri*, respectively. Different aphid species were used because of the aphid host selectivity, but clover to clover SbDV-MD6 transmission efficiency was very similar for both vector species (data not shown). The SbDV-MD6 isolate was maintained in the same white clover from the point of collection until each passaging experiment, with additional infected source plants generated solely by the vegetative propagation of runners. This clover isolate was used as an initial inoculum source for pea and soybean lines, and these infected pea and soybean plants were then used as sources for next serial transmissions, continuing through eight passages, or until transmission was lost. The transmission efficiency of each passage was determined by the percentage of successfully infected pea or soybean plants.

**Figure 1:**
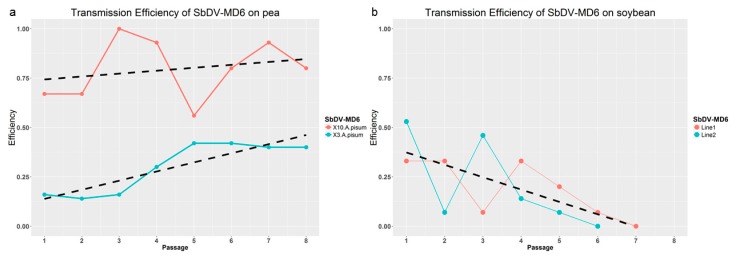
The transmission efficiency of Soybean Dwarf Virus (SbDV)-MD6 on pea and soybean lines. (**a**) transmission efficiency of SbDV-MD6 on peas with two different numbers of aphid for inoculation; (**b**) transmission efficiency of SbDV-MD6 on soybean, two independent experiments were conducted with indicated trend.

In peas, initial SbDV-MD6 inoculations were done with 10 aphids for each pea seedling. The transmission efficiency was 67% at first passage, and remained fairly consistently above 80% after the second passage with the exception of passage 5 (Figure 1a). In a second serial transmission with three aphids per plant, improvement was observed after the third passage, and the transmission efficiency was maintained at around 40% until the end of the serial passage experiment (Figure 1a). In pea serial passages, the transmission efficiency had a statistically significant increase with passages when using three pea aphids per plant. In contrast to serial passages in peas, SbDV-MD6 transmission efficiency in soybean generally deceased with serial transmission. The transmission efficiency on soybeans was more variable than the transmission efficiency on peas, and after five or six passages in soybeans, SbDV-MD6 transmission was reduced to the point where successful transmission did not occur in this experiment set up (Figure 1b).

### 3.2. Symptom Development and Virus Titer in Alternative Hosts

**Figure 2:**
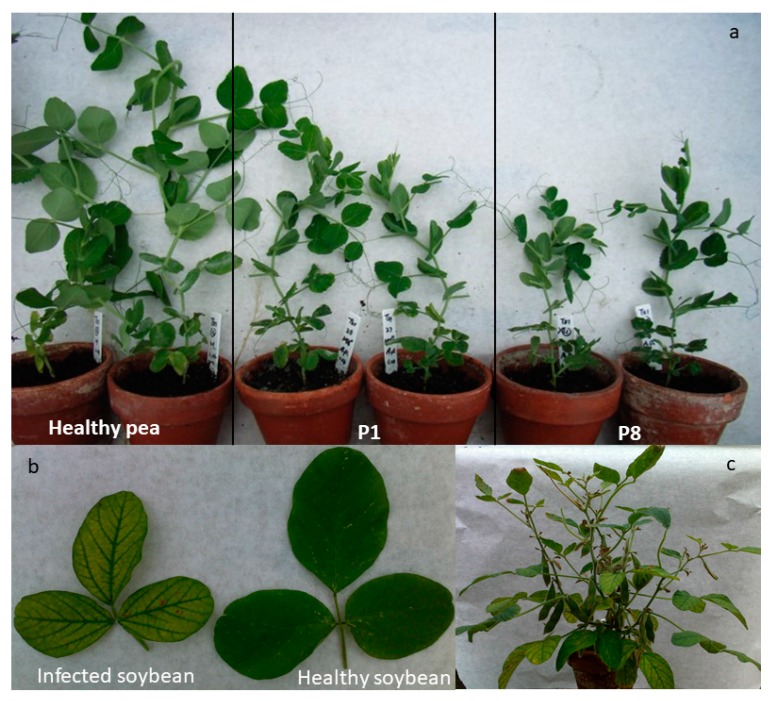
Symptom comparison of healthy and SbDV-MD6 infected pea and soybean plants. (**a**) SbDV-MD6 infected showed increased dwarfing and stunted growth from passage 1 to passage 8 when compared to healthy control plants at 25 days post-inoculation; (**b**) SbDV-MD6 infected soybeans are yellowing compared with healthy soybeans; (**c**) SbDV-MD6 infected soybeans showed yellowing and leaf elongation, but no dwarfing at 40 d.p.i.

**Figure 3:**
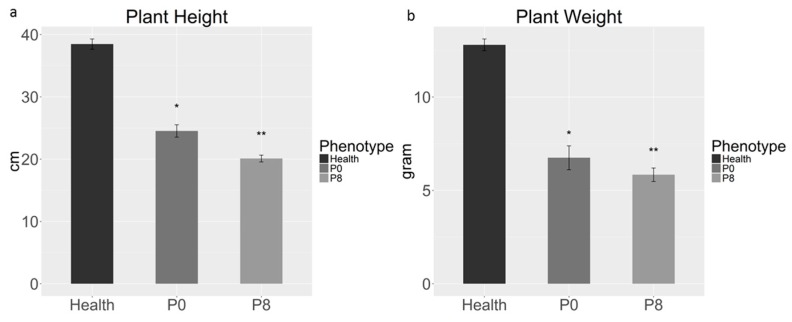
Statistical comparison of height and fresh weight of infected peas indicates significant differences between passages 0 and 8. Bars with one or two asterisks are significantly different from control plants at *p* < 0.05 (*) and 0.01 (**), respectively. Error bars represent the standard error of the mean based on two independent experiments.

The level of symptom development was determined by observing visible symptoms and by measuring plant height and fresh weight in each passage. The symptoms of SbDV-MD6 infected peas did not appear until at least 20 d.p.i. All infected pea plants from various passages (P1-P8) were significantly shorter and had less fresh weight than the healthy controls (Figure 2a). Despite the fact that SbDV-MD6 is a yellowing strain, there was no more chlorosis in infected pea leaves than in healthy controls (Figure 2a). Plants in the final passage (P8) of SbDV-MD6 in pea were also significantly shorter, and had reduced fresh weight compared to those of the first passage (Figure 3). Therefore, symptom severity did increase slightly following serial transmissions in peas. Symptoms on soybeans were less dramatic: only moderate chlorosis of infected leaves (Figure 2b). No significant dwarfing or reduced biomass was observed in soybeans at any time (Figure 2c). Symptom severity of SbDV-MD6 infected soybeans did not show observable differences with continued passages.

**Figure 4:**
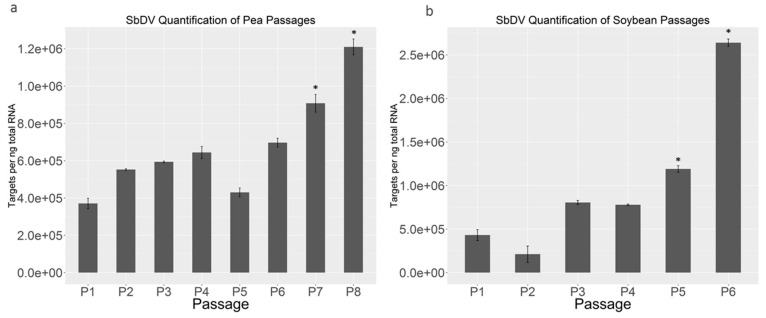
Quantitative real-time PCR (qRT-PCR) for virus quantification of SbDV-MD6 during serial transmissions in pea-passage (**a**) and soybean-passage (**b**) lines at 30 d.p.i. Statistical significance levels (determined by one-way ANOVA, *p* < 0.05) indicated by asterisks.

Quantitative real-time-PCR was used to assess viral titers of SbDV-MD6 in pea and soybean at each passage (Figure 4). Virus titers were determined in each of two independent experiments for both pea and soybean. All positive samples were tested individually with three replications. Using an SbDV-MD6 in vitro transcript as a template, the standard curve was established with serial dilutions in healthy plant extracts. The mean titer of first passages in peas was 3.7 × 10^5^ target molecules per nanogram (ng) of total RNA extraction. The titers of SbDV-MD6 increased slightly with continued passage on peas (Figure 4a), and P7 and P8 were significantly different from P1, suggesting increased SbDV-MD6 concentration in peas following sequential transmission in the new pea host. The mean virus titer in the initial passages of soybean was 4.3 × 10^5^ targets molecules per ng of total RNA extraction, and the virus titers for subsequent passages continued to increase (up to 2.55 × 10^6^ targets), suggesting increasing virus titers with each subsequent transmission. Overall, there was a more dramatic increase of viral titers resulting from sequential soybean transmissions compared to pea. It indicated that SbDV-MD6 had an approximately 6-fold increase in viral titer by the end of both serial passage experiments.

### 3.3. Clover Back Inoculation Assays

Pea and soybean adapted SbDV-MD6 were transmitted back to white clover to evaluate the potential trade-off effects associated with adaptation to new hosts. The back-inoculation transmission efficiency decreased significantly after passage 1 in pea hosts (Table 1). However, the pea adapted SbDV-MD6 populations never lost the ability to re-infect clover, even after eight passages in peas. Back inoculations from soybean-adapted populations to healthy clover seedlings were only attempted after the first and final passages. The data suggested that the soybean adapted SbDV-MD6 back inoculation transmission efficiency might be reduced after serial transmission; however, the differences were not statistically significant (Table 1). The reduction in clover infection efficiency by soybean adapted SbDV-MD6 suggested that SbDV-MD6 populations encountered the reduced fitness in clover by either reduced transmission ability and/or reduced infection capacity in clover.

### 3.4. Sequence Analysis of SbDV-MD6 in Serial Passages

To determine what molecular changes had occurred in the viral population following the infection of new hosts, the complete genomes of SbDV-MD6 in pea and soybean passage lines were sequenced. First, the complete genome of original population in clover was sequenced as a reference for comparison. The completed consensus sequence of original white clover SbDV-MD6 included 5862 nucleotides (Genbank accession No. JN674402), and the genome organization of the five major ORFs was consistent with all other SbDV-Y strains [13]. In the pea lines, the complete genome of first passage, two middle passages (PsP2, JN674404; PsP4, JN674405), and last passage (PsP8, JN674406) were sequenced. The same approach was taken with the soybean lines (GmP1, JN674407; GmP4, JN674408; GmP6, JN674409). As a control, the last passage in the clover-to-clover transmission line, done in parallel with the pea lines as a positive transmission control, was also sequenced. 

**Figure 5:**
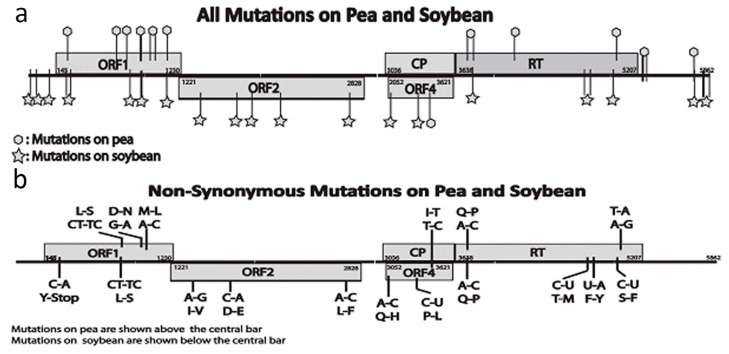
Locations of mutations that occurred on pea and soybean during serial transmissions. A schematic diagram of the host adapted SbDV-MD6 genomes with genes labeled are shown. (**a**) represents all mutations observed in SbDV-MD6 from pea (hexagons above genome) and from soybeans (stars below genome); (**b**) represents non-synonymous mutations observed in SbDV-MD6 from pea and from soybeans with nucleotide changes shown closer to the schematic, and amino acid changes shown distally from the schematic.

For serial transmission on peas by *A. pisum*, the total number of mutations observed was 26 in all passages, and there were a total of 17 consistent synonymous and non-synonymous mutations that occurred in both passage lines and were maintained through to the end of passaging (Figure 5a,b). There were four synonymous mutations (nucleotide positions 282, 737, 745, and 1153) and three non-synonymous mutations (940, 1066, 1081) in ORF1. Within the CP gene, there was only one synonymous mutation. However, the synonymous mutation at 3461 on CP changed a codon from isoleucine to threonine in the nested ORF4 gene. In the RT ORF5, which is important for aphid transmission, two non-synonymous mutations (nucleotide positions 3706 and 5007) and two synonymous mutations (nucleotide positions 3830 and 4232) occurred. In addition, there were also four mutations (nucleotide positions 5284, 5285, 5288 and 5627) in the 3’-UTR region. The *d*_N_ values of all pairwise comparison of ORFs were lower than corresponding *d*_S_ values, with the average 0.31 for ORF1, and 0.22 for RT ORF5. Other ORFs were undergoing very weak selection. These *d*_N_/*d*_S_ values are consistent with a population under weak selection. Only two non-synonymous mutations were observed in the clover-to-clover control transmission line.

In serial transmission on soybeans by *N. bakeri*, there was a total of 28 mutations observed in all passages; of these, 23 were consistent mutations (Figure 5a,b). In contrast to peas, many of these mutations (11) changed amino acids. Two non-synonymous mutations (nucleotide positions 309, 940 and 941) occurred in ORF1, three (nucleotide positions 1452, 1796, and 2757) occurred in ORF2, two (nucleotide positions 3090 and 3392) in ORF4 (these mutations had no effect on the amino acid sequence of the coat protein), and four (nucleotide positions 3706, 4810, 4837, and 5050) on the RT ORF5. There were three synonymous mutations (nucleotide positions 282, 810 and 1153) in ORF1. Three mutations were found (nucleotide positions 9, 64 and 126) in the 5′-UTR, and four mutations were also found (nucleotide positions 5627, 5775, 5776 and 5782) in the 3′-UTR. Unexpectedly, the mutation at nucleotide position 309 changed the reading frame from a tyrosine codon to a stop codon. The *d*_N_/*d*_S_ rate of consistent mutations was 0.26 for both ORF1 and ORF2 genes, and 0.59 for RT ORF5 gene. In the clover control line, no non-synonymous mutations were observed, and only two synonymous mutations (nucleotide position 2027 and 5095) were found after four serial transmissions.

For the back inoculation of passage 6 and 8 in pea transmission lines, the sequences of the mutation regions were checked. Most of the mutations that were identified in serial transmissions described above reverted to the same as sequences in the original clover, including three non-synonymous mutations (nucleotide position 1066, 1081, and 5007). There are only three synonymous mutations (nucleotide position 282, 3830 and 4232) preserved when the viral populations were transmitted from pea back into the clover. For SbDV-MD6 populations back inoculated from soybean to clover, all mutations reverted to the original clover sequence with the exception of two mutations in the ORF5 gene (nucleotide position 3706 and 4810).

## 4. Discussion

In initial characterization studies, a number of SbDV isolates (VA-20, MD6, MD8, MD10, MD11 and MD16) from the eastern United States were transmitted to soybean [9]. However, for all of these strains, attempts at serial transmissions were unsuccessful. Typically, the SbDV strain introduced into soybean could be transmitted once or twice, but transmission efficiency decreased dramatically with serial passage on soybean until transmission eventually failed. In order to investigate this observation, a single well characterized isolate was chosen for controlled passage studies on both soybean and pea. The isolate used for this study was SbDV-MD6, a Y strain isolate collected from Prince George’s county in Maryland [9]. Multiple SbDV-MD6 infected white clover were collected and identified from the edge of a soybean field, but there were no SbDV symptoms observed in the adjacent soybeans. Both *A. pisum* and *N. bakeri* were present in the clover surrounding the soybean fields, but neither of these aphids was observed feeding on soybeans. Based on this information, the natural history of SbDV-MD6 was assumed to be limited to a clover infection, transferred from clover to clover by *A. pisum* and/or *N. bakeri*. There were no peas (*Pisum sativum* L.) observed anywhere within a 1 km radius of the originally infected white clover.

In the serial transmission experiments, SbDV-MD6 was transmitted to peas with high efficiency (67%) from the beginning of the passaging when using 10 *A. pisum* per pea plant, and maintained a high level of transmission efficiency thereafter. When three aphids were used for the inoculation, the most dramatic improvement of transmission efficiency was observed after the third transmission (Figure 1a). It would appear that the efficiency of transmission to pea was so good that the only way to see the subtle improvement with continued passage was to limit the inoculum load presented at each passage. qRT-PCR suggested that viral titers in pea passage lines were increasing by the second passage and significantly increasing by P7 (Figure 4a). Symptom severity on infected peas increased with serial transmissions, as the later passages of infected peas became significantly shorter and smaller than the initial passage (Figure 2a). The increases in titer, disease symptom severity, and transmission efficiency all suggested that SbDV-MD6 was adapting to the peas. 

In contrast to the pea serial transmission lines, the transmission efficiency on soybeans was initially low, and it decreased with serial transmission on soybean, despite the fact that a significantly higher number of aphids were used in soybean inoculations. The competency of the vector could be a factor in the poor transmission efficiency. However, *N. bakeri* typically transmitted SbDV from infected clover to healthy clover with an average of 66% efficiency (data not shown) and transmitted from infected clover to soybean with 33–53% efficiency following the first or second passage to soybean (Figure 1b). Fluctuations in transmission efficiency might result from inconsistent aphid feeding behavior on the non-preferred host (soybean). Although soybean was not the preferred host, *N. bakeri* was able to feed on soybean to acquire and transmit the virus, and were observed to survive and feed on soybean for the duration of the 5-day inoculation access period. Furthermore, if the aphid vector competency was the sole factor limiting transmission efficiency, one would expect that the transmission efficiency would remain relatively constant. The qRT-PCR data indicated that SbDV-MD6 could be adapting to soybeans as a host during the serial transmission process, since viral titers in soybeans improved dramatically after the third passage. Overall, SbDV-MD6 populations in soybeans demonstrated an approximately 6-fold increase in viral concentration based on qRT-PCR (Figure 4b). An alternative explanation for the reduction of transmission efficiency in soybean passage lines was the possibility that host adaptive mutations in viral populations passaged on soybean had deleterious effects for the aphid vector transmission. Aphid transmission of luteoviruses requires very specific interactions between aphids and viruses [23]. Although it would seem likely that transmission efficiency was not necessarily related to the host adaptation, it was not out of the realm of possibility that host adaptive mutations could have a deleterious effect on a separate biological process, aphid transmission.

Sequence analysis of SbDV-MD6 genomes recovered from the pea and soybean host serial passages might suggest possible mechanisms for the observed increases in virus titers along with the reduction of virus transmission in soybean. The non-synonymous mutations that change amino acids would be expected to affect biological functions. In pea passages, three non-synonymous mutations were located in ORF1. This protein is directly involved in viral replication and is likely to interact with host proteins. An additional non-synonymous mutation was found in ORF4, which has been identified as the movement protein, another likely source of host directed selection. The CP ORF3 had no non-synonymous mutations identified in any of passaged lines for both pea and soybean, suggesting the conserved function of CP, where only synonymous mutations were identified. Two more non-synonymous mutations were found in the RTP ORF5, which had been suggested to also contribute to tissue specificity within the plant host [24]. In addition, it is important to remember that SbDV-MD6 may also be undergoing selection for transmission by *A. pisum* as the viral population was serially transmitted. There were four additional mutations in the 3’-UTR of pea-passaged SbDV-MD6. The 3’-UTR is related to replication initiation [25], although this portion of the genome is rather variable amongst SbDV isolates. 

In soybean passage lines, the SbDV-MD6 *d*_N_/*d*_S_ values were higher than the *d*_N_/*d*_S_ values observed in pea passage lines. Both ORF1 and ORF2 had been undergone purifying selection pressure during the new host adaptation. Interestingly, the mean value 0.59 of RT ORF5 in soybean was significantly higher than the mean value 0.22 in pea passages. This suggests that the RT ORF5 genes making the host shift from clover to soybean encountered less purifying selection pressures than the SbDV-MD6 populations making the shift from clover to pea. As ORF5 is one of key factors for luteovirus vector transmission and systemic infection [26], higher genetic diversity in the soybean host may cause trade-off effects during the host shifting [27]. The more dramatic improvements in viral titer but lower transmission efficiency over the course of serial passage on soybean would support this hypothesis. The sites where amino acids changed in SbDV-MD6 soybean passages were mainly located on replication and RT coding regions. Just as with the pea serial passage, SbDV-MD6 passaged on soybean may also be undergoing selection for transmission by *N. bakeri*. The mutations in the replication related proteins (ORF1 and ORF2) and the 5′ UTR (important replication control sequence) could easily be related to host selection pressures, but it is hard to imagine the exact function and effects of mutations in the C-terminal of the RTP. 

Previous research suggests that RTP is related to vector transmission [12], and it is possible that the RTP mutations are related to selection for *N. bakeri* transmission. However, in this work, the evidence indicated that transmission efficiency by *N. bakeri* was decreasing in soybean serial transmissions. Therefore, there might be additional functions for the RTP, such as host tissue specificity and intercellular transport [24,26,28,29,30,31], and the mutations adapting to plants have a trade-off effect on vector transmission. It was interesting to note that SbDV-MD6 accumulated non-synonymous mutations in the RTP serially passaged on peas. When these pea-adapted virus populations were back inoculated into clover again, most of the mutations (including all of the non-synonymous mutations) reverted to the original clover sequence, further suggesting that these mutations were likely related to host adaptation. It was also interesting that one of the non-synonymous changes in soybean passage lines introduced a stop codon in ORF1, a critical protein for viral replication. However, all of the sequencing was performed directly on RT-PCR products, so it would be impossible to determine if the mutations were not present in a minority of the population without a more detailed cloning analysis. In addition, an internal methionine codon was available for the initiation of translation, just downstream from the introduced stop codon. The significant improvement in viral titers suggested that at least a functional level of ORF1 protein products must be available to the viral population.

On the surface, the reduction of aphid transmissibility in SbDV-MD6 soybean serial transmissions does not make sense. Certainly any viral population making such an adjustment in a field setting would be rapidly out competed by neighboring transmissible populations, regardless of replication efficiency within the host. However, it is important to remember that the format of the serial transmissions done here was heavily biased towards generating viral populations that were strongly adapted to the host plants. Although luteoviruses only infect host phloem cells, the viral populations were exposed to host related selection pressures in every infected cell, from the very beginning to the very end of the passage experiment. In contrast, the aphid vector itself constitutes a temporal selection pressure that is never actually associated with a replicating viral population. Under these conditions, if there is a mutation that is beneficial to the virus in the new host regime but deleterious to aphid transmission, this mutation would become dominant in the population even if the advantage in the host was minimal and the cost to transmissibility drastic. 

These experiments might lead to speculation that soybeans were not really a viable alternative host for SbDV-MD6. A host that selects for a non-transmissible population would certainly seem to be an evolutionary dead end. However, that point of view would be flawed because of the host selection biased limitations of the experimental design. The conditions used here and in many other experimental systems do not necessarily reflect the reality for viruses in the field. In the field, particularly on perennial pasture hosts such as clover, aphids are a near constant presence. As such, the selection pressures for maintaining aphid transmissibility would almost certainly be stronger, and have a greater influence on the eventual makeup of the viral population. The role of near-continuous aphid transmission as a selection pressure on clover isolates of SbDV to adapt to new aphid vectors species or to new plant hosts has yet to be determined. However, this work does demonstrate that the compact nature of viral genomes may in certain cases constrain the amount of allowable variability, as mutations that would be advantageous to one phase of the viral life cycle (replication of movement) may be deleterious to another phase of the viral life cycle (transmission). This may be especially true for multifunctional viral proteins and overlapping reading frames.

## Figures and Tables

**Table 1:** The transmission efficiency of back-inoculating clover with SbDV-MD6 from pea using *A. pisum* and *N. bakeri*, and soybean passages using *N. bakeri* only.

SbDV-MD6		Passage ^1^ (Plants Infected/Total Inoculated)							
Hosts	No. of Vectors	1	2	3	4	5	6	7	8
Pea Line 1	20 *N. bakeri*	4/6 *	2/6	4/6	2/6	0/6	1/6	1/6	1/6
	20 *A. pisum*	4/6 *	- ^3^	-	1/6	0/6	0/6	2/6	1/6
Pea Line 2	20 *N. bakeri*	5/6 *	-	-	2/6	1/6	-	2/6	0/6
	20 *A. pisum*	4/6 *	-	-	1/6	0/6	-	1/6	1/6
Soybean Line 1 ^2^	30 *N. bakeri*	3/10	-	-	-	-	0/10	-	-
Soybean Line 2 ^2^	30 *N. bakeri*	2/10	-	-	1/10	0/10	-	-	-

^1^ Infected source plants used for aphid acquisition of SbDV-MD6 correspond to infected plants shown in Figure 1; ^2^ For soybean lines, only infected plants from the first and last passages were tried; ^3^ Data not available; * Significant difference (*p* < 0.05).

## Supplementary Materials

The following are available online at www.mdpi.com/1999-4915/9/5/155/s1, Figure S1: Experimental design for the serial transmission of SbDV-MD6 in pea or soybean plants, Figure S2: Sequencing strategy for determination of full-length consensus sequences of SbDV-MD6.
