# Supplementary material for: Host Adaptation of Soybean Dwarf Virus Following Serial Passages on Pea (Pisum sativum) and Soybean (Glycine max)
Source: Viruses. 2017 Jun 21;9(6):155. doi: 10.3390/v9060155 (PMC5490830; doi:10.3390/v9060155)
Supplement: Supplementary file 1 [file viruses-09-00155-s001.pdf]

Supplemetary material: Host Adaptation of Soybean Dwarf Virus Following Serial Passages on Pea (Pisum sativum) and Soybean (Glycine max)

Bin Tian, Frederick E. Gildow, Andrew L. Stone, Diana J. Sherman, Vernon D. Damsteegt and William L. Schneider

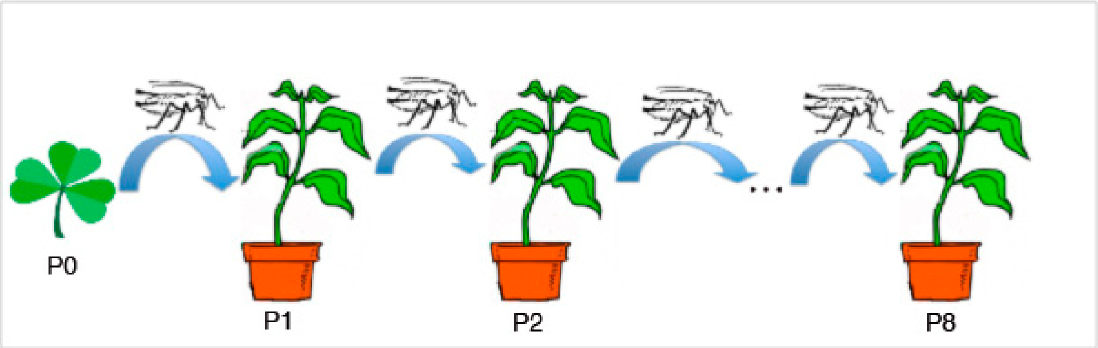

Figure S1. Experimental design for the serial transmission of Soybean Dwarf Virus (SbDV)-MD6 in pea or soybean plants. The original infected clover (P0) was used as the source plant. Infected plants in the first passage (P1) were pooled to serve as the source for the next transmission. Each passage line was continued through eight passages (P8), or until transmission failed.

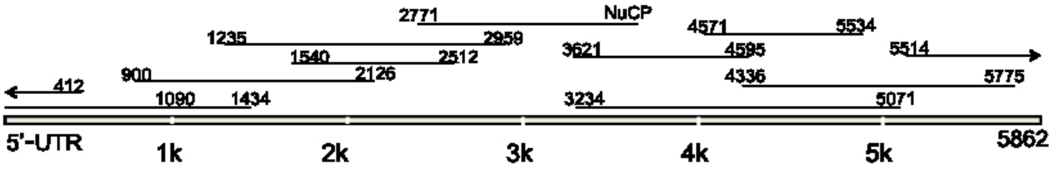

Figure S2. Sequencing strategy for determination of full length consensus sequences of SbDV-MD6. PCR fragments (thin lines) were generated using the primers (named above PCR fragments. Two additional primers (1090R, 4336F) were used to determine internal sequences of larger PCR fragments.
